# Supplementary material for: A High Performance Triboelectric Nanogenerator Based on MXene/Graphene Oxide Electrode for Glucose Detection
Source: Materials (Basel). 2023 Jan 15;16(2):841. doi: 10.3390/ma16020841 (PMC9863351; doi:10.3390/ma16020841)
Supplement: Supplementary file 1 [file materials-16-00841-s001.zip › materials-2153441-supplementary.pdf]

materials

A High Performance Triboelectric Nanogenerator Based on MXene/Graphene Oxide Electrode for Glucose Detection

Wei Yang [1,2,†], Xu Cai [1,2,†], Shujun Guo [1,2], Long Wen [1,2], Zhaoyang Sun [1,2], Ruzhi Shang [1,2], Xin Shi [1,2], Jun Wang [1,2], Huamin Chen [1,2,3,*] and Zhou Li [3,*]

1 College of Materials and Chemical Engineering, Minjiang University, Fuzhou 350108, China; yangwei@mju.edu.cn (W.Y.); 16655015937@163.com (X.C.); 2527@mju.edu.cn (S.G.); 18381141672@163.com (L.W.); xiatiansun2022@163.com (Z.S.); shangruzhi1102@163.com (R.S.); shixin202212@163.com (X.S.); wangjun2@mju.edu.cn (J.W.)
2 Fujian Key Laboratory of Functional Marine Sensing Materials, Minjiang University, Fuzhou 350108, China
3 Beijing Institute of Nanoenergy and Nanosystems, Chinese Academy of Sciences, Beijing 100083, China
* Correspondence: chenhuamin@mju.edu.cn (H.C.); zli@binn.cas.cn (Z.L.)
† These authors contributed equally to this work.

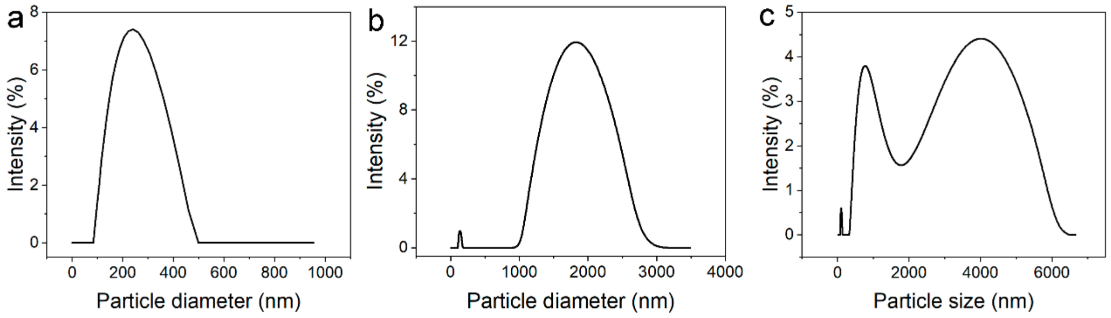

Figure S1. The DLS measurement of different GO nanosheets. (a) GO (small size), (b) GO (medium size), (c) GO (large size),.

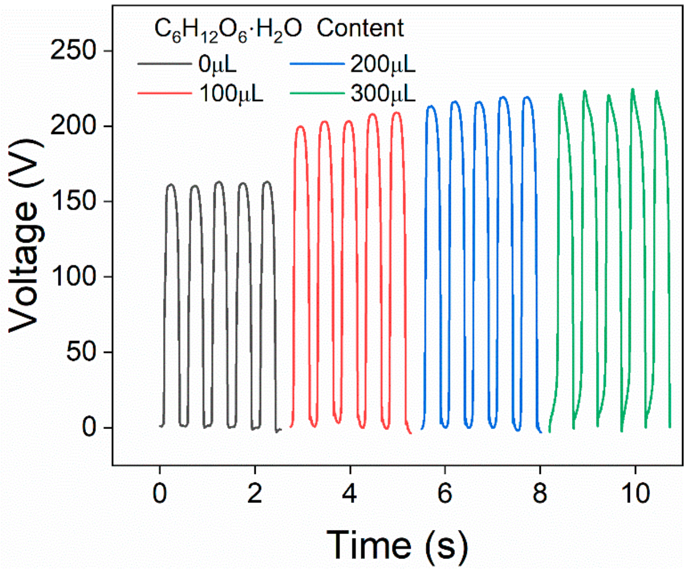

Figure S2. The relationship between the output voltage of MXene-based TENG and the glucose concentration.

Table S1. The comparison with previous works.

Year	Materials	Target	Detection limit and work range	Output voltage	Ref.
2013	Nano-TiO_2	Catechin	5 μM	~ 5V	S1
2013	Cu/PDMS	Glucose	0.1–1 mM	17 V	S2
2016	Al/Gelatin	Glucose	200 μM–2 mM	500 V	S3
2022	Enzymes	Glucose	0.0056 mM	3 V	S4
2022	MXene/bac-terial	Zn^{2+} Cu^{2+}	1 μM 10-300 μM	10.9 V	S5
	MXene/GO	Glucose	100 μM	258.8 V	Our work

Reference:

[S1] Lin, Z.; Xie, Y.; Yang, Y.; Wang, S.; Zhu, G.; Wang, Z., Enhanced Triboelectric Nanogenerators and Triboelectric Nanosensor Using Chemically Modified TiO2 Nanomaterials. *ACS Nano* **2013**, 7(5), 4554–4560.

[S2] Zhang, H.; Yang, Y.; Hou, T.; Su, Y.; H, C.; Wang, Z., Triboelectric nanogenerator built inside clothes for self-powered glucose biosensors. *Nano Energy* **2013**, 2(5), 1019-1024.

[S3] Chang, T.; Wang, C.; Chen, C.; Li, Y.; Hsu, C.; Chang, H.; Lin, Z., Controlled synthesis of Se-supported Au/Pd nanoparticles with photo-assisted electrocatalytic activity and their application in self-powered sensing systems. *Nano Energy*, **2016**, 22, 564-571.

[S4] Zhao, T.; Fu, Y.; Sun, C.; Zhao, X.; Jiao, C.; Du, A.; Wang, Q.; Mao, Y.; Liu, B., Wearable biosensors for real-time sweat analysis and body motion capture based on stretchable fiber-based triboelectric nanogenerators. *Biosens. Bioelectron.* **2022**, 205, 114115.

[S5] Wang, E.; Cao, Y.; Bai, Y.; Gai, Y.; Shan, Y.; Li, Q.; Jiang, T.; Feng, H.; Li, Z., A triboelectric nanosensor based on ultra-thin MXene composite paper for heavy metal ion detection. *J. Micromech. Microeng.* **2022,** 32, (4), 044003.
